# The role of leptin in indirectly mediating “somatic anxiety” symptoms in major depressive disorder

**DOI:** 10.3389/fpsyt.2022.757958

**Published:** 2022-07-15

**Authors:** Yue Zhu, Yange Wei, Jia Duan, Jianing Li, Ran Zhang, Jiaze Sun, Pengshuo Wang, Zhuang Liu, Jing Lv, Shengnan Wei, Xiaowei Jiang, Fei Wang, Yanqing Tang

**Affiliations:** ^1^Department of Psychiatry, The First Affiliated Hospital of China Medical University, Shenyang, China; ^2^Brain Function Research Section, The First Affiliated Hospital of China Medical University, Shenyang, China; ^3^China Medical University and Queen’s University of Belfast Joint College, China Medical University, Shenyang, China; ^4^School of Public Health, China Medical University, Shenyang, China; ^5^Department of Psychiatry, Corning Hospital, Shenzhen, China; ^6^Department of Radiology, The First Affiliated Hospital of China Medical University, Shenyang, China; ^7^Department of Gerontology, The First Affiliated Hospital of China Medical University, Shenyang, China

**Keywords:** drug-naïve major depressive disorder, first-degree relatives, leptin, somatic anxiety symptoms, mediation analysis

## Abstract

**Background:**

Leptin is a multifunctional hormone secreted from adipose tissue, which plays a core role in regulating energy intake and expenditure. Evidence has demonstrated that leptin receptors are located in brain areas involved in emotional processing, and major depressive disorder (MDD) is characterized by dysfunction of emotional processing. Taken together, these features suggest that leptin may play a potential role in the pathophysiology of MDD. However, the precise roles of leptin in modulating depressive symptoms in MDD remain unclear.

**Methods:**

Participants [18 drug-naïve MDD patients, 15 unaffected first-degree relatives of MDD patients (FDR-MDD), and 40 healthy controls] completed clinical assessments and provided blood samples for measurement of leptin levels. We evaluated the effect of leptin on clinical status (MDD or FDR-MDD) and symptomatic dimensionalities of MDD using mediation analysis.

**Results:**

We found that leptin was increased in MDD patients and this only predicted “somatic anxiety” symptoms. Furthermore, leptin was a significant and indirect mediator of the association between clinical status (MDD or FDR-MDD) and “somatic anxiety” symptoms.

**Conclusion:**

Our finding that leptin was a significant and indirect mediator of clinical status (MDD or FDR-MDD) and “somatic anxiety” symptoms suggests that leptin may indirectly affect somatic depressive symptoms in MDD. Our findings may provide a theoretical basis for novel clinical interventions in MDD.

## Background

Major depressive disorder (MDD) is one of the most prevalent and disabling mental disorders worldwide (1). Genetic epidemiological research has indicated that compared with the general population, people with one first-degree relative with a mood disorder are approximately 2.8 times more likely to suffer from MDD (2). However, despite this increased genetic risk, most first-degree relatives of individuals with MDD (FDR-MDDs) do not develop MDD. Importantly, examination of FDR-MDDs may be useful for determining whether there are candidate markers of vulnerability in individuals with risk genes of MDD.

There is a bidirectional relationship between depressive disorder and obesity, such that the presence of one disorder increases the risk of developing the other (3). Furthermore, patients with obesity and their first-degree relatives frequently experience depression, anxiety, and other psychiatric disturbances (4, 5). Leptin is secreted by adipocytes in peripheral tissues and plays a core role in regulating energy intake and expenditure (6). Circulating leptin can permeate the blood–brain barrier (BBB) to exert its central effects *via* participating in synaptic activity, neuronal morphology, and neuronal development in the central nervous system (7, 8), and circulating leptin is partly from the human brain (9). Leptin receptors participate in emotional processing and exhibits high expression in the cortex, amygdala, and hippocampus (10, 11). The distribution of leptin signaling in the brain is related to emotional and cognitional processes, which has sparked an increased interest in the role of leptin in mood disorders (12–14). However, studies have provided conflicting results with both high and low levels of circulating leptin in depressed patients (15–17), suggesting that circulating leptin levels are influenced by age, sex, body mass index (BMI), and treatment with antidepressants (18, 19).

The relationship between depressive and anxiety symptoms of MDD and leptin remains unclear. A cross-sectional study indicated that metabolic syndrome was associated with the severity of depressive symptoms and the prevalence of depression (20). In patients with metabolic syndrome, leptin is positively associated with somatic depressive symptoms but not total depressive symptoms (21). Moderate-severe anxiety symptoms are associated with high serum leptin levels in patients with type 2 diabetes (22). Higher phobic anxiety scores are associated with increased levels of serum leptin in women with diabetes (23). These above studies focused on the relationship between leptin and depressive symptoms in patients with metabolic syndrome. MDD studies have reported conflicting findings on the relationship between leptin levels and the severity of depressive or anxiety symptoms in MDD, as these studies have found both positive and negative relationships, as well as no relationship whatsoever (24, 25). Higher leptin levels were associated with an atypical MDD subtype, but not with overall MDD or the typical subtype (26). These mixed results may be attributed to different symptomatic profiles of MDD; additionally, most studies have aimed to study total depressive symptoms of MDD without elucidating the symptom dimensions more commonly associated with leptin. Hence, it may be helpful to understand the role of leptin in MDD *via* the multidimensional clinical character of MDD.

In this study, we aim to investigate whether drug-naïve MDD patients and FDR-MDDs exhibit dysregulation of leptin, to identify whether there is a specific symptomatic dimension affected by leptin in MDD, and to explore whether leptin is an indirect mediator in this specific symptomatic dimension.

## Materials and methods

### Participants

Participants included drug-naïve patients with MDD (*n* = 18), FDR-MDDs (*n* = 15), and healthy controls (HCs) (*n* = 40), all aged between 13 and 45 years. Drug-naïve MDD patients were recruited from 2014 to 2017 in the Department of Psychiatry at the First Affiliated Hospital of China Medical University and Shenyang Mental Health Center. Sixteen MDD patients were in a depressive state, and two MDD patients were in a remissive state. FDR-MDDs were all first-degree relatives of patients presenting with MDD at the Department of Psychiatry at the First Affiliated Hospital of China Medical University and Shenyang Mental Health Center. HCs were recruited from the local community *via* advertisements. All participants provided written consent that was approved by the Ethics Committee of China Medical University.

Participants with MDD were diagnosed by two trained psychiatrists individually and were included if they met the following criteria: (1) they fulfilled the Schedule for Affective Disorders and Schizophrenia for School-Age Children (KSADS-PL) criteria if younger than 18 years; (2) they fulfilled the Structured Clinical Interview DSM-IV criteria for MDD if 18 years or older; and (3) they had no comorbid diagnosis of psychosis or bipolar disorder, and no history of psychotropic medications. FDR-MDD participants were all first-degree relatives of individuals with MDD who did not meet the criteria for any DSM-IV Axis-I disorder. HCs were individuals who did not have a current or previous history of Axis-I disorders and did not have any first-degree relatives with a history of Axis-I disorders. The severities of depression and anxiety of all participants were assessed using the 17-item Hamilton Depression Rating Scale (HAMD-17) and the Hamilton Anxiety Rating Scale (HAMA). The multidimensional characteristics (“somatic anxiety,” “psychic anxiety,” “core depressive,” and “anorexia”) of the HAMD-17 determined *via* factor analysis have been deemed to be useful for better understanding psychopathological dimensions of MDD (27). The “somatic anxiety” psychopathological dimension of MDD in the HAMD-17 includes somatic anxiety, hypochondria, early insomnia, middle insomnia, late insomnia, general somatic symptoms, and gastrointestinal symptoms.

The present study was approved by the Institutional Review Board of China Medical University and was performed in accordance with the Declaration of Helsinki. All experiments and methods were performed in accordance with approved guidelines and regulations. Demographic and clinical details are presented in Table 1.

**TABLE 1:** Demographic and clinical characteristics of HC, HR-MDD, and drug-naïve MDD.

	HC	GHR-MDD	MDD	*F*/χ^2^ value	*P*-value
*n*	40	15	18		
Age	25.22 (4.84)	30.00 (7.46)	23.89 (7.55)	4.517	0.014
Gender, female%	47.50%	53.30%	77.80%	4.684	0.098
BMI	21.83 (4.04)	23.11 (3.90)	21.77 (3.49)	0.663	0.518
Duration, months	−	−	14.24 (20.05)	−	−
First episode, yes	−	−	83.30%	−	−
**HAMD**					
Somatic anxiety	0.33 (0.69)	0.60 (1.18)	5.59 (4.68)	31.12	<0.001
Psychic anxiety	0.13 (0.52)	0.27 (0.80)	5.18 (8.01)	10.77	<0.001
Core depressive	0.13 (0.34)	0.13 (0.52)	4.06 (2.93)	47.74	<0.001
Anorexia	0.15 (0.43)	0.20 (0.56)	1.00 (1.17)	9.38	<0.001
HAMD total	0.65 (1.27)	1.13 (2.23)	14.88 (10.34)	48.39	<0.001
**HAMA**					
HAMA total	0.57 (1.55)	1.33 (2.16)	13.06 (11.55)	29.56	<0.001

Data are mean (SD) or %. HAMD, Hamilton Depression Rating Scale; HAMA, Hamilton Anxiety Rating Scale.

### Determination of plasma leptin levels

Blood collection was carried out according to standardized protocols, with samples taken between 10:00 a.m. and 3:00 p.m. Participants should not have consumed food for at least 2 h before the blood collection. EDTA was used as an anticoagulant. Plasma samples were centrifuged for 10 min at 2,000 rpm and were stored at −80°C until further analysis. A Human Premixed Multi-Analyte Kit (R&D Systems, Inc., Minneapolis, MN, United States) with a Human Magnetic Luminex Assay was used to measure plasma leptin levels. Samples were magnetically labeled using a human magnetic premixed microparticle cocktail of antibodies (Kit Lot Number L120614). The assay was performed in duplicate according to the manufacturer’s directions, and intra- and inter-assay coefficients of variation were <10% for leptin. Detailed information on this method can be found in Supplementary Material.

### Statistical analyses

We separated the participants into three groups (HCs, FDR-MDDs, and MDD patients). Group effects on demographic characteristics (age, gender, and BMI) and clinical characteristics (duration of illness, first episode, and HAMD and HAMA scores) were examined using one-way analyses of variance (ANOVAs) or Chi-square tests. Leptin concentrations were analyzed using a one-way analysis of covariance (ANCOVA), with age, gender, and BMI as covariates. *Post hoc* analyses were performed among the HC, FDR-MDD, and MDD groups using a general linear model. Bonferroni correction was used for multiple comparisons.

We used partial correlation to analyze the correlation between leptin levels and clinical symptoms, age, gender, and BMI as covariates in the MDD group. We then used multiple stepwise regression analysis to examine the effects of leptin on clinical symptom scores with potential confounding factors (age, gender, and BMI) in the MDD group. Based on these results, a mediation analysis was used to explore whether leptin (as a mediator variable) potentially influenced the association between clinical status-MDD or FDR-MDD (causal variable) and clinical symptoms (outcome variable). For the mediation analysis, the PROCESS procedure for SPSS Version 3.2 (written by Andrew F. Hayes, Ph.D.^1^) was used, with a 5,000 bias-corrected bootstrap sample for significance testing. We summarized mediators using means, standard deviations (SD), and 95% confidence interval (CI). Significance was set at *p* < 0.05 (two-tailed) for all tests. All analyses were performed using SPSS.

## Results

### Demographic and clinical characteristics

There were significant differences in age (*p* = 0.014) among the HC, FDR-MDD, and MDD groups. There were no significant differences in gender or BMI (*p* > 0.05) among the HC, FDR-MDD, and MDD groups. The effects of diagnosis on HAMD (“somatic anxiety,” “psychic anxiety,” “core depressive,” “anorexia,” and total scores) (27) and HAMA scores were significant among the HC, FDR-MDD, and MDD groups (all *p*-values < 0.001; Table 1).

### Comparison of plasma leptin levels

**FIGURE 1:**
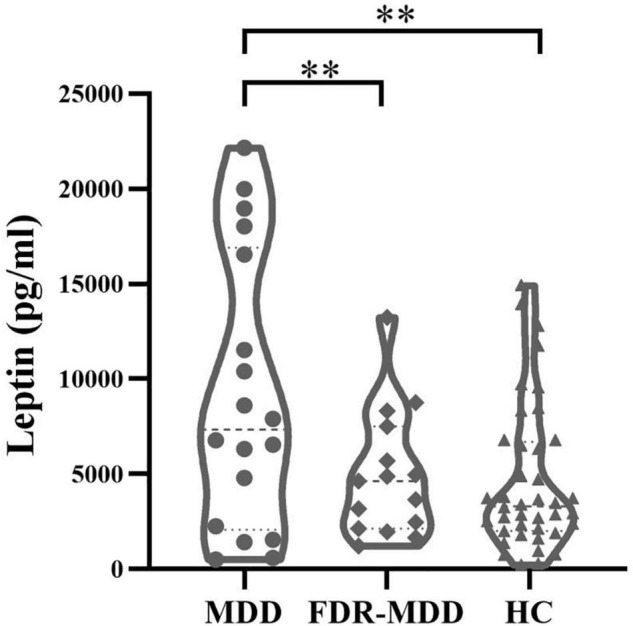
Comparison of plasma leptin levels by groups. Higher plasma leptin levels in drug-naïve MDD (9,163.11 ± 7,184.88 pg/ml) compared with FDR-MDD (4,956.07 ± 3,320.44 pg/ml, *p* = 0.003) and HC (4,633.3 ± 3,836.81 pg/ml, *p* = 0.008) after Bonferroni correction. **p < 0.01.

After controlling for age, gender, and BMI, significant group effects were observed in leptin levels in the three-group analysis (*p* = 0.004). *Post hoc* analysis revealed significantly higher leptin levels in the MDD group compared with those in FDR-MDD (*p* = 0.003) or HC (*p* = 0.008) group after Bonferroni correction (*p_*Bonferroni*_* = 0.017), but there was no significant difference in leptin levels between the FDR-MDD and HC groups (Figure 1).

### Relationship between leptin levels and clinical symptoms

**FIGURE 2:**
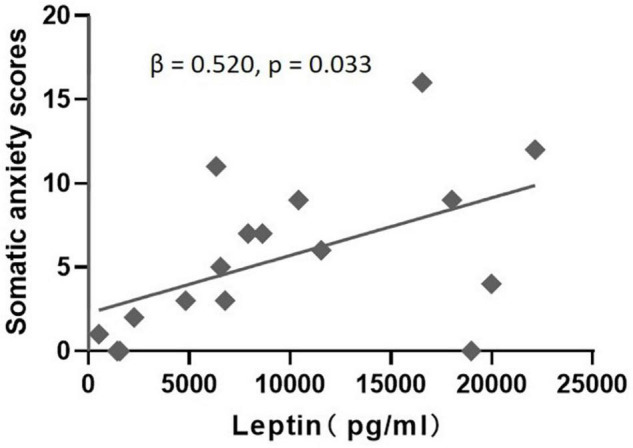
Multiple regression analysis to examine the effect of leptin on somatic anxiety scores after accounting for age, gender, and BMI in the MDD group.

In the MDD group, correlation analysis identified a significant positive correlation between leptin levels and “somatic anxiety” scores on the HAMD (*r* = 0.550, *p* = 0.024), but no significant correlation between leptin levels and scores for “psychic anxiety,” “core depressive,” “anorexia,” or total scores on the HAMD and HAMA (Table 2). Regression analyses further confirmed that plasma leptin levels were only significantly positively associated with “somatic anxiety” scores on the HAMD (β = 0.520, *t* = 2.355, *p* = 0.033) in the MDD group after controlling potential confounding factors (age, gender, and BMI) (Figure 2).

**TABLE 2:** Correlations between levels of leptin and clinical symptoms in MDD.

	HAMD					HAMA total
	Somatic anxiety	Psychic anxiety	Core depressive	Anorexia	Total	
*r*-value	0.550	0.230	0.425	−0.162	0.388	0.417
*p*-value	0.024*	0.374	0.089	0.535	0.124	0.095

*Correlation coefficients statistically significant at p < 0.05.

### Mediated moderation analysis

**FIGURE 3:**
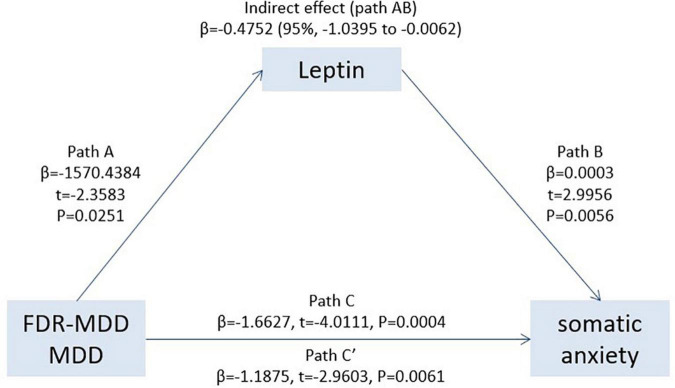
Leptin significantly mediated the association between clinical status (MDD or FDR-MDD) and “somatic anxiety,” providing further evidence that there was an indirect way to influence patient “somatic anxiety” by leptin. Path C represents the variance in clinical state associated with “somatic anxiety,” and Path C′ represents the association between clinical status and “somatic anxiety” after taking into account leptin as a mediator. Path AB in the mediation effect and is significant at *p* < 0.05 based on confidence intervals from bias-corrected bootstrapping of 5,000 samples.

Based on the results of the multiple regression analyses, we established a mediation model and found that leptin (Path AB, β = −0.4752; 95% CI: −1.0395 to −0.0062) significantly mediated clinical status in “somatic anxiety” as measured by the HAMD. As shown in Figure 3, clinical status was significantly related to leptin levels (Path A, β = −1570.4384, *t* = −2.3583, *p* = 0.0251), and leptin was significantly positively associated with “somatic anxiety” on the HAMD (Path B, β = 0.0003, *t* = 2.9956, *p* = 0.0056). The total effect (effect of clinical status on “somatic anxiety”) was also significant (Path C, β = −1.6627, *t* = −4.0111, *p* = 0.0004). After accounting for leptin as a mediator, the direct effect of clinical status on “somatic anxiety” was significant (Path C′, β = −1.1875, *t* = −2.9603, *p* = 0.0061). Leptin played an indirect effect (Path AB, β = −0.4752), which accounted for 28.58% (Path AB/Path C) of the total effect.

## Discussion

To the best of our knowledge, our present study is the first to investigate leptin levels in drug-naïve MDD patients, FDR-MDDs, and HCs to determine if there is a specific symptomatic dimension in MDD influenced by leptin levels and to further explore the precise role of leptin in mediating this specific symptomatic dimension of MDD. We found that leptin was increased in participants with MDD. Subsequently, we found leptin predicted “somatic anxiety” symptoms in MDD, and leptin could be a significant and indirect mediator in the association between clinical status (MDD or FDR-MDD) and “somatic anxiety” symptoms.

### Dysregulation of leptin in major depressive disorder

We found that leptin was increased in the drug-naïve MDD group but failed to find the same change in the FDR-MDD group, suggesting that increased leptin levels in MDD may indicate that elevated leptin may play an important pathophysiological role in MDD. Consistent with our finding, previous studies demonstrated elevated circulating leptin levels in patients with MDD (15, 28, 29). Circulating leptin as an adiposity negative signal could transmit peripheral energy homeostatic information to the brain. Peripheral hyperleptinemia related to central leptin resistance could explain the reason for anomalous appetite/weight changes in MDD. Central leptin resistance could occur at several levels, including debilitated transport of leptin across the BBB, the diminished function of the leptin receptor, and defects in leptin signal transduction (30). However, other studies reported reduced plasma leptin in MDD patients with a normal BMI (16, 17); this is consistent with rats or mice models of depression, which exhibited low circulating leptin levels (31, 32). Animal model data support the hypothesis that leptin insufficiency may underlie depression-like behavioral deficits. The conflicted findings in circulating leptin in humans may be related to the emergence of leptin insufficiency in a subpopulation of depressed patients. Another interpretation may be that circulating leptin levels are influenced by multiple factors. Sexual dimorphism may affect leptin levels, with leptin levels being higher in females than in males (33, 34). A meta-analysis by Cao et al. indicated that males who expressed lower adiponectin and leptin levels had a higher likelihood of developing MDD (35). In their meta-analysis, they also found that there was no significant difference in leptin levels between MDD subjects and HCs (SMD = 0.13; 95% CI: −0.06, 0.31; *p* = 0.170); however, there was high heterogeneity for leptin (*I*^2^ = 91.8%, *p* < 0.001). After excluding six Asian studies, significantly higher levels of leptin were found in MDD subjects compared to those in HCs. The geographic location of participants may contribute to the heterogeneity of reported leptin levels (35). One previous study has posited that dysfunction of central leptin signaling, rather than the absolute concentration of leptin, may be more associated with effects on mood (13). Paz-Filho et al. systematically reviewed that there are possible therapeutic uses of leptin in conditions where leptin levels were normal, low, or high, and also suggested that a better understanding of the physiological roles of leptin may contribute to the development of leptin-based treatments for depression (36). Therefore, further investigations are needed to better elucidate the mechanisms of leptin in modulating the neurobiological substrates of MDD and in the potential application of leptin levels in the clinical application of MDD.

### Leptin indirectly mediates depressive symptoms in major depressive disorder

In the present study, we found a positive correlation between leptin and “somatic anxiety” but not for other symptoms in the MDD group. “Somatic anxiety” includes somatic anxiety, sleep disturbances, general somatic symptoms, gastrointestinal symptoms, and hypochondria. A previous study indicated that increased leptin levels were accompanied by sleep disturbances (37). The irregularity of the sleep-wake rhythm would affect energy homeostasis, and the energy homeostasis could be regulated by leptin (38). Leptin expression in the gastric mucosa could be associated with gastrointestinal symptoms (39). Our finding is also consistent with previous research which indicated that there is a significantly positive association between leptin and somatic depressive symptoms after adjusting for relevant confounding factors such as age, gender, BMI, insulin resistance, and inflammatory factors (21). Unfortunately, the mechanisms of the relationship between leptin and somatic depressive symptoms remain unknown. Based on the neuroendocrine functions of emotion regulation of leptin, mediation analysis showed that leptin was an indirect mediator in the association between clinical status and “somatic anxiety” symptoms. Similar to any application of regression analysis, our mediation analysis proves that the model is correctly specified but does not generate evidence that establishes causality (40). Therefore, we cannot bluntly arrive at a conclusion that leptin plays a causal role in somatic anxiety symptoms in MDD, due to other factors that may influence the relationship between leptin and “somatic anxiety” symptoms. Leptin may provide feedback information on the nutritional status *via* integrated regulation of energy balance (41). In this study, all participants were informed to not consume food for at least 2 h prior to the blood collection to reduce short-term effects of eating on leptin. Peripheral leptin was affected by longer periods of fasting and overfeeding but was not changed immediately in the following food within 3 h (42). Additionally, nutritional status is linked to depression. However, it is difficult to conclude a causal relationship in human subjects since it is unclear whether depressed patients changing their healthy or unhealthy dietary habits may increase the risk of depression. A previous study also found that leptin levels were associated with chronic stress conditions (depressive mood and social isolation), while this association was not influenced by other lifestyle factors (smoking status, alcohol consumption, physical activity, and low income) (43). The associations among depression, unhealthy lifestyle factors (e.g., chronic stress conditions and unhealthy dietary habits), and leptin are complex. Chronic stress activates the hypothalamic–pituitary–adrenal (HPA) axis (44) and induces low-grade inflammation (45). Inflammation can reduce leptin signals to the central nervous system that influence depression and leptin may modulate HPA function (12, 46). Leptin modulates energy homeostasis *via* gut-brain circuits (47) and also regulates reward processing of food by modulating dopaminergic mesolimbic systems (48). MDD patients may be more vulnerable to dysfunctional food-reward processing, which may trigger stress-induced eating, and this phenomenon may be related to altered leptin levels in MDD. Therefore, there seems to be a complex interacting relationship among the following: MDD-unhealthy lifestyle factors (e.g., chronic stress conditions and unhealthy dietary habits), HPA dysfunction, inflammation, leptin dysregulation, mood disturbances, and MDD. Although our findings suggest that leptin may play a role in mediating somatic anxiety symptoms in MDD, the mechanisms of leptin-related somatic anxiety symptoms in MDD remain unclear.

### Limitations

Our present study had some limitations. Firstly, we selected drug-naïve MDD patients to minimize the influence of treatments, resulting in an extremely small sample size that may have limited the generalizability of our findings. Therefore, we performed power analyses of ANOVA and correlation analyses in the MDD group. ANOVA achieved the power of 0.67 and correlation analyses achieved the power of 0.51. Another limitation of the present study is that this was a cross-sectional study; therefore, subsequent developments in the included FDR-MDDs remain unknown. A longitudinal study of FDR-MDDs with long-term follow-ups is required to make such determinations, with comparisons between individuals who do and do not develop MDD, allowing for the possibility of developing a better mechanism to determine the genetic susceptibility of MDD.

## Conclusion

In summary, we found that leptin was increased in MDD patients and that elevated leptin may play an important pathophysiological role in MDD. Additionally, we found a correlation between leptin and “somatic anxiety” symptoms in MDD patients, and leptin was found to be a significant and indirect mediator between clinical status (MDD or FDR-MDD) and “somatic anxiety” symptoms, suggesting that leptin plays an indirect effect in somatic depressive symptoms in MDD. Taken together, our findings may provide a theoretical basis for novel clinical interventions for treating MDD.

## Data Availability Statement

The raw data supporting the conclusions of this article will be made available by the authors, without undue reservation.

## Ethics statement

All participants provided written consent that was approved by the Ethics Committee of China Medical University. Written informed consent to participate in this study was provided by the participants’ legal guardian/next of kin.

## Author contributions

YZ, YW, FW, and YT designed the study. RZ, JS, PW, JL, SW, and XJ have collected participants. YZ, JD, JNL, and ZL did the analysis plan. YZ drafted the manuscript. All authors read, contributed, and approved the final manuscript.

## Conflict of Interest

The authors declare that the research was conducted in the absence of any commercial or financial relationships that could be construed as a potential conflict of interest.

## Publisher’s Note

All claims expressed in this article are solely those of the authors and do not necessarily represent those of their affiliated organizations, or those of the publisher, the editors and the reviewers. Any product that may be evaluated in this article, or claim that may be made by its manufacturer, is not guaranteed or endorsed by the publisher.

## Footnotes

1
www.afhayes.com

## Abbreviations

MDD: major depressive disorder
FDR-MDDs: first-degree relatives of MDD
BBB: blood–brain barrier
HC: healthy control
KSADS-PL: Schedule for Affective Disorders and Schizophrenia for School-Age Children
HAMD-17: 17-item Hamilton Depression Rating Scale
HAMA: Hamilton Anxiety Rating Scale
ANOVA: one-way analysis of variance
BMI: body mass index
ANCOVA: one-way analysis of covariance
SD: standard deviation
CI: confidence interval
HPA: hypothalamic–pituitary–adrenal
