# Supplementary material for: The role of leptin in indirectly mediating “somatic anxiety” symptoms in major depressive disorder
Source: Front Psychiatry. 2022 Jul 15;13:757958. doi: 10.3389/fpsyt.2022.757958 (PMC9337242; doi:10.3389/fpsyt.2022.757958)
Supplement: Supplementary file 1 [file Table_1.DOCX]

**Supplementary** **Material**

**Supplementary Methods**

Measurement of plasma leptin levels

Blood samples (5 ml) were collected and centrifuged at 2,000 rpm for 10 minutes. The plasma was stored in aliquots at -80°C for leptin measurements. Samples were magnetically labeled using a human, magnetic, premixed, microparticle cocktail of antibodies (Kit Lot Number L120614). 50μL of the microparticle cocktail and 50μL of the standard or sample were added to each well of a microplate. Plates were then placed on a horizontal orbital microplate shaker set at 800 ± 50 rpm. After washing, 50μL of a diluted biotin-antibody cocktail was added to each well and the plates were incubated. After washing, 50μL diluted Streptavidin-PE was added to each well, and the plates were incubated. After washing, the microparticles were suspended by adding 100μL of wash buﬀer to each well. The plates were then incubated for 2 minutes on the shaker set at 800 ± 50rpm. The final sample was read within 90 minutes using a Luminex MAGPIX technology (SN, USA). Duplicate readings for each standard and sample were averaged, and the average blank Median Fluorescence Intensity (MFI) was subtracted. A standard curve was generated for each cytokine to convert the MFI into the corresponding leptin relative concentration. The sensitivity of the assay for leptin was 104.940 pg/ml (minimum detectable level).
